# Soluble programmed death ligand 1 as prognostic biomarker in non-small cell lung cancer patients receiving nivolumab, pembrolizumab or atezolizumab therapy

**DOI:** 10.1038/s41598-024-59791-0

**Published:** 2024-04-18

**Authors:** Sinne Søberg Brun, Torben Frøstrup Hansen, Sara Witting Christensen Wen, Christa Haugaard Nyhus, Lisbeth Bertelsen, Anders Jakobsen, Torben Schjødt Hansen, Line Nederby

**Affiliations:** 1 Department of Oncology, Vejle Hospital-University Hospital of Southern Denmark, Beriderbakken 4, 7100 Vejle, Denmark; 2 Department of Regional Health Research, Faculty of Health Sciences, University of Southern Denmark, J. B. Winslowsvej 19, 3, 5000 Odense, Denmark; 3 Department of Biochemistry and Immunology, Vejle Hospital-University Hospital of Southern Denmark, Beriderbakken 4, 7100 Vejle, Denmark

**Keywords:** sPD-L1, NSCLC, Advanced disease, Anti-PD-1/anti-PD-L1 treatment, Prognosis, Biochemistry, Biological techniques, Cancer, Immunology, Biomarkers, Medical research

## Abstract

Many studies have focused on the prognostic role of soluble programmed death ligand 1 (sPD-L1) in non-small cell lung cancer (NSCLC), but outcomes are ambiguous and further investigations are needed. We addressed the matter by studying sPD-L1 in baseline samples and in longitudinal samples taken prior to three subsequent cycles of anti-PD-1/anti-PD-L1 treatments. Eighty patients with NSCLC were enrolled. Median sPD-L1 level at baseline was 52 pg/mL [95% confidence interval (CI) 49–57]. In patients treated with pembrolizumab and nivolumab, the concentration of sPD-L1 remained rather stable throughout treatment. In contrast, sPD-L1 rose by 50-fold following the first cycle of atezolizumab therapy. We found the baseline level of sPD-L1 to be related to overall survival (OS) after two years of follow-up in simple Cox analysis (p = 0.006) and multiple Cox Regression, hazard ratio 1.02 (95% CI 1.00–1.03) (p = 0.033). There was no association between sPD-L1 and tissue PD-L1 expression, overall response rate, or progression free survival. In conclusion, sPD-L1 measured in baseline serum samples may be associated with OS in NSCLC patients receiving anti-PD1/anti-PD-L1 treatment. Importantly, the results signify that further research is warranted to explore the clinical utility of sPD-L1 in patients treated with anti-PD-L1.

## Introduction

Lung cancer is the leading cause of cancer-related death around the world^1^. In recent years, immunotherapy has significantly improved the treatment of this disease and in particular the immune-checkpoint inhibitors against programmed cell death protein 1 (PD-1)/programmed death ligand 1(PD-L1) have improved the survival of non-small cell lung cancer (NSCLC) patients^2–5^. T cells, B cells, monocytes, dendritic cells and Natural Killer cells express the trans-membrane glycoprotein PD-1^6^. In healthy tissue binding between PD-1 and its ligands PD-L1 and programmed death ligand 2 (PD-L2) cause downregulation of the immunological activity thus resulting in self-tolerance and reduced tissue damage during clearing of infections, however malignant cells exploit this essential pathway. Upregulation of PD-L1 in the tumor causes down-regulating of the T cells’ anti-tumor activity^7^. By blocking the interaction between PD-L1 and PD-1, anti-PD-1/anti-PD-L1 therapies are able to restore the immune response against the cancerous cells^7^.

Although anti-PD-1/anti-PD-L1 therapies have shown promising therapeutic effects against advanced NSCLC and locally advanced NSCLC, a large proportion of patients do not benefit from this treatment and selecting the right patient for therapy remains challenging^8,9^. Presently, evaluating expression level of PD-L1 in the tumor tissue by immunohistochemistry (IHC) guides treatment decisions, however, PD-L1 is not a reliable predictive biomarker^10^. While association between high PD-L1 expression and response to anti-PD-1/anti-PD-L1 treatment and overall survival has been published for both NSCLC and other malignancies, other studies have shown efficacy of the same antibodies to be independent of PD-L1 expression^8^. Reasons for this incongruity may be intra-tumor and inter-tumor heterogeneous levels of PD-L1 as well as temporal variation of PD-L1 expression, which are not necessarily detected in biopsies^11^. Moreover, the lack of a standardized IHC assay makes it difficult to do a direct comparison of these studies^10^. Therefore, in pursuit of pinpointing the right patients for anti-PD-1/PD-L1 therapies additional biomarkers are needed. In this context, there has been focus on both immunological biomarkers and cell-free DNA, and although several promising candidates have been described, more evidence are needed for these to become clinically applicable^12^.

A soluble form of PD-L1 (sPD-L1) is gaining interest as a potential biomarker in cancer and studies have reported sPD-L1 levels to be an adverse prognostic marker in several malignancies^13^. Allegedly, the pool of sPD-L1 arise from both alternative splicing of PD-L1 and proteolytic cleavage of membrane bound PD-L1, while PD-L1 on the surface of extracellular vesicles are also part of the total. Although studies in lung cancer demonstrated an association between PD-L1 on tumor cells and level of sPD-L1^14,15^, others were unable to demonstrate such relationship and as a results suggest that both tumor cells and extratumoral inflammatory background are contributors to the level of sPD-L1^16^. Hence, the origin of sPD-L1 is not fully established and interestingly neither is the function although studies have shown that sPD-L1 is able to bind PD-1 on T-cells causing both inhibition and apoptosis. Furthermore, sPD-L1 is thought to lower the efficacy of anti-PD-1/anti-PD-L1 therapies as it can bind anti-PD-L1, thus limiting the concentration of antibody at the tumor site, and it may bind to PD-1 thus reducing epitopes available for anti-PD-1 treatments^17–19^.

The potential role of sPD-L1 as a biomarker in NSCLC has been investigated without a definitive conclusion, however most publications advocate that this protein is of prognostic value^15,16,20–22^. Obviously, in NSCLC patients undergoing anti-PD-1/anti-PD-L1 therapies efforts have been made to determine whether sPD-L1 are predictive of response, but so far results are ambiguous and more investigations are needed to elucidate this matter.

In this retrospective study, we measured serum sPD-L1 levels in patients with advanced NSCLC who received nivolumab, pembrolizumab or atezolizumab therapy. The aim was to assess the relationship between sPD-L1 and tumor PD-L1 expression, the clinical characteristics, the response to treatment and patient outcome. Furthermore, we investigated the dynamics of sPD-L1 in serum collected at baseline and immediately prior to the three subsequent cycles of anti-PD-1/anti-PD-L1 treatment.

## Methods

### Patients and study design

Between 1 August 2017 and 31 January 2020, we prospectively enrolled 80 patients with advanced or recurrent NSCLC candidate for anti-PD-1/anti-PD-L1 therapy at the Department of Oncology, Vejle Hospital, Denmark. Seventy-four patients received pembrolizumab, three patients received nivolumab, and three patients received atezolizumab. All patients had blood samples collected at baseline and immediately before the second, third, and fourth treatment. Inclusion criteria were patients with a histologically confirmed diagnosis of NSCLC, minimum 18 years, and candidates for anti-PD-1/anti-PD-L1 therapy. An exclusion criterion was other concurrent experimental treatment within fourteen days of enrollment. The clinical information was last updated 14 July 2022. Nivolumab was administered intravenously every two weeks at dosages of 3 mg/kg, while pembrolizumab and atezolizumab was given every 3 weeks at 200 mg and 1200 mg, respectively. The treatment was continued until clinical or radiographic progression, unacceptable toxicity, or death. The effects of pembrolizumab, nivolumab, or atezolizumab on NSCLC was assessed by the physicians and radiologists, according to Response Evaluation Criteria In Solid Tumors, version 1.1^23^. Progression free survival (PFS) and overall survival (OS) were defined as time from therapy start until disease progression and death, respectively.

All methods were performed in accordance with the relevant guidelines and regulations. The study was approved by the Regional Committee on Health Research Ethics in Southern Denmark (ID: S-20170063) and the Danish Data Protection Agency (ID: 18/33058) and was conducted in accordance with the Declaration of Helsinki. All participants provided written informed consent.

### Sample collection

Blood samples were collected from enrolled patients at baseline and immediately prior to the subsequent three treatments. Whole blood was collected into 10 mL BD Vacutainer clot activator tubes (BD, NJ, USA) using the BD Vacutainer^®^ Safety-Lok™ Blood Collection Set with pre-attached holder (BD, USA). Samples were centrifuged at 2000×g at 20 °C for 10 min within 4 h of sampling and the serum was stored in aliquots of 1 mL at – 80 °C until use.

### Measurement of sPD-L1

Soluble PD-L1 was measured in all serum samples using the Simoa^®^ PD-L1 Discovery Kit (Quanterix, Lexington, Massachusetts, USA) and the Simoa HD-1 analyzer™ (Quanterix). Samples were thawed, centrifuged, diluted × 20 in Sample Diluent, and analyzed using a Neat protocol as recommended by the manufacturer. Controls (two prepared from the PD-L1 calibrator and Calibrator Diluent and three serum samples) and the calibrator were analyzed in duplicates. Measured in-house, the Intra-assay variation was < 10% while inter-assay variation was < 16%. Moreover, sPD-L1 was measured in serum from atezolizumab-treated patients using the Human/Cynomolgus Monkey PD-L1/B7-H1 Quantikine ELISA kit in a 1:2 dilution (R&D Systems, Minneapolis, Minnesota, USA).

### Statistical analysis

Blood samples except baseline samples were excluded from two patients treated with atezolizumab, because measured sPD-L1 concentrations were extremely high compared to all other values. We report median values with a 95% confidence interval (CI). The Wilcoxon rank sum test/Mann–Whitney U test was used for comparison of median values. The Kaplan–Meier method was used to illustrate survival curves and the log-rank test to test for differences between the curves. Cox regression was used for single marker and multiple marker testing. All statistical calculations were performed using the GraphPad Prism version 9.5.1 software (GraphPad Software, Boston, Massachusetts, USA). p values < 0.05 were considered significant.

## Results

### Patient characteristics

Eighty patients were included in the study (Table 1). The median age was 70 (range 53–84). The majority of the patients had stage IV disease, nine were stage III, and one patient had stage II disease. Four patients were in performance status (PS) 2 when they initiated treatment. Thirty-two patients were PS 1 and 44 patients were PS 0. Anti-PD-1/anti-PD-L1 therapy was started as first line treatment in 50 (63%) patients, whereas 30 (38%) patients received one or more lines of treatment before the anti-PD-1/anti-PD-L1 therapy. Three patients had hypoalbuminemia when enrolled. At the time of analyses, 81% of the patients had died. The median PFS was 5.8 months [95% confidence interval (CI) 2.9–7.8] and median OS was 15.5 months (95% CI 8.9–20.2).

**Table 1:** Patient characteristics and sPD-L1 measured in baseline samples. Percentages do not always add up to 100% due to rounding of data. p-values are based on the nonparametric Mann–Whitney U test. Bold p-values refer to a significant difference. *sPD-L1* soluble programmed death ligand 1, *COPD* Chronic obstructive pulmonary disease, *SCC* Squamous cell carcinoma.

Parameter	N = 80 (%)	sPD-L1 (95% CI)	p-value
Sex			
Male	34 (43)	60 (50–66)	**0.015**
Female	46 (58)	49 (45–55)	
Age, median 70			
≥ 70	43 (54)	54 (48–63)	0.487
< 70	37 (46)	51 (48–56)	
Stage			
II–III	10 (13)	58 (49–66)	0.272
IV	70 (88)	52 (48–57)	
Performance status			
0	44 (55)	52 (48–57)	0.501
1–2	36 (45)	53 (48–63)	
Smoking status			
Never	2 (3)		
Previous	55 (69)	51 (45–56)	0.068
Active	20 (25)	62 (48–73)	
Unknown	3 (4)		
COPD			
Yes	24 (30)	52 (45–60)	0.949
No	55 (69)	54 (48–59)	
Unknown	1 (1)		
Histology			
Adenocarcinoma	56 (70)	54 (48–59)	0.203
SCC	17 (21)	50 (37–53)	
Other	7 (9)		
PD-L1 tissue			
< 50%	17 (21)	51 (41–64)	0.672
≥ 50%	63 (79)	53 (49–59)	
Line of treatment			
First	50 (63)	56 (50–62)	**0.034**
Second or third	30 (38)	49 (45–54)	

In Table 1 the potential association between baseline sPD-L1 levels and clinic characteristics are given. Patients receiving anti-PD-1/anti-PD-L1 therapy as first line treatment had a median sPD-L1 significantly higher than patients receiving the therapy as second or third line treatment (56 pg/mL vs. 49 pg/mL, p = 0.034). Moreover, males had significantly higher levels of sPD-L1 at baseline than females did (60 pg/mL vs. 49 pg/mL, p = 0.015). There was no association between tissue PD-L1 expression and sPD-L1. Median sPD-L1 was 51 pg/mL (95% CI 41–64) for patients with tissue PD-L1 expression > 50% and 53 pg/mL (95% CI 49–59) for patients with tissue PD-L1 expression < 50%, p = 0.672. The baseline level of sPD-L1 measured in the three hypoalbuminemic patients were in the upper tertile of all eighty baseline sPD-L1 results obtained.

### Longitudinal measure of sPD-L1

**Figure 1:**
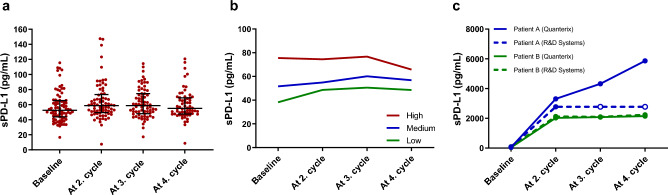
Concentration of soluble programmed death ligand 1 (sPD-L1) in non-small cell lung cancer patients. (**a**) Concentration of sPD-L1 at the four sampling time points. Medians and interquartile ranges are illustrated. (**b**) Patients were divided in tertiles based on the sPD-L1 baseline level (red = high (n = 27), blue = medium (n = 27), green = low (n = 26)). Graphs illustrate the baseline level and dynamics of sPD-L1 in the subsequent samples in the three groups. (**c**) Dynamics of sPD-L1 in the two patients receiving atezolizumab measured using assays from two individual manufacturers. Open circles represent values above detection limit of the assay.

Serum samples were available for analysis from all patients at baseline (100%), 90% before second treatment, 89% before third treatment, and 79% before the fourth treatment. Figure 1a shows sPD-L1 measured in samples collected at baseline and immediately prior to the subsequent three treatments. Median sPD-L1 at baseline was 52 pg/mL (95% CI 49–57). When grouping the patients in tertiles based on their baseline levels of sPD-L1, it was evident that sPD-L1 remained rather stable throughout treatment (Fig. 1b). The same result was seen when looking separately at the patients receiving first-line therapy and patients receiving second or third line treatment (Supplementary Fig. 1). In contrast, in the two patients treated with atezolizumab, the baseline sPD-L1 levels were 49 pg/mL and 41 pg/mL, but after the first treatment, these increased to 3312 pg/mL and 2030 pg/mL, respectively, and sPD-L1 remained strongly elevated during the subsequent samples (Fig. 1c). Assessing sPD-L1 from the atezolizumab treated patients using an alternative assay (R&D Systems) instigated comparable measures (Fig. 1c).

### sPD-L1, treatment efficiency and prognoses

The overall response rate (RR) for the cohort was 28% (22/79). The treatment course for one patient was not evaluable due to a shift to chemotherapy after only one cycle of immunotherapy. There was no association between RR and sPD-L1 based on a grouping in three, 32% (8/25) for sPD-L1 low, 19% (5/27) for sPD-L1 medium, and 33% (9/27) for sPD-L1 high, p = 0.751, respectively.

**Figure 2:**
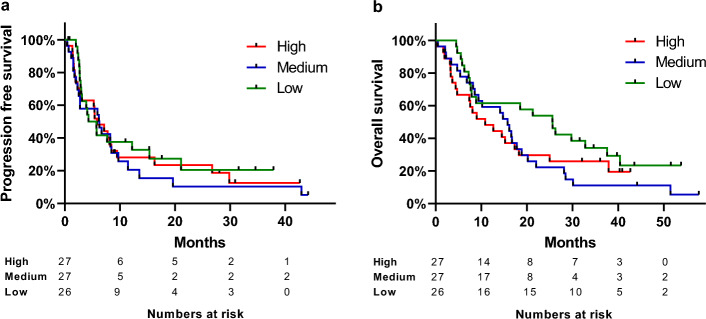
Progression free survival (p = 0.681) (**a**) and overall survival (p = 0.195) (**b**) according to baseline levels of soluble programmed death ligand 1 (sPD-L1). The patients (N = 80) were divided in tertiles based on the sPD-L1 baseline level (red = high, blue = medium, green = low).

For all patients, regardless of line of treatment, the relationships between sPD-L1 and PFS and OS are illustrated in Fig. 2. The median PFS was 5.8 months (95% CI 2.3–9.5) for sPD-L1 high, 6.2 months (95% CI 2.1–8.9) for sPD-L1 medium, and 4.2 months (95% CI 2.7–12.2) for sPD-L1 low. For the sPD-L1 high group median OS was 10.9 months (95% CI 4.4–25.0), 15.7 months (95% CI 8.2–20.2) for sPD-L1 medium, and 25.6 months (95% CI 7.7–34.6) for sPD-L1 low. When analyzing the dataset obtained over the entire follow-up period there were no significant relationships with either PFS (p = 0.681) nor OS (p = 0.195) when grouped according to tertiles. Assessing baseline sPD-L1 as a numeric parameter, according to Cox Regression, did not alter the conclusion regarding PFS, HR 1.01 (95% CI 0.99–1.02), p = 0.168, but resulted in a significant relationship with OS, HR 1.01 (95% CI 1.00–1.03), p = 0.034. The results from the simple and multiple Cox Regression analyses are shown in Table 2. Based on the simple testing, sample size, and number of events we chose PS, smoking status, and sPD-L1 for the multivariate testing. Performance status was the only parameter that remained of significant importance concerning OS, HR 1.69 (95% CI 1.00–2.87), p = 0.049. According to clinical guidelines, lung cancer patients may receive pembrolizumab, nivolumab, or atezolizumab for up to two years. When using two years as the follow-up period, there was still no significant relationship with either PFS (p = 0.680) nor OS (p = 0.090) when grouped according to tertiles. If assessing sPD-L1 as a numeric parameter in a simple cox regression analysis, there was no association with PFS, HR 0.97 (95% CI 0.92–1.01), p = 0.178, however a significant relationship with OS was evident, HR 1.02 (95% CI 1.01–1.03), p = 0.006 (Table 2). In multivariate analysis, adjusting for PS and smoking status, sPD-L1 remained a prognostic factor for OS, HR 1.02 (95% CI 1.00–1.03), p = 0.033. Moreover, performance status was also a significant influence in the multivariate analysis, HR 1.86 (95% CI 1.05–3.32), p = 0.033.

**Table 2:** Simple and multivariate testing of association between sPD-L1, relevant patient characteristics, and overall survival (N = 80). The analyses were performed for both the complete follow-up period and for the 2-year follow-up period. Bold p-values refer to a significant difference. *sPD-L1* soluble programmed death ligand 1, *COPD* Chronic obstructive pulmonary disease, *SCC* Squamous cell carcinoma.

	Complete follow-up period				2-year follow-up period			
	Simple test		Multivariate test		Simple test		Multivariate test	
	HR (95% CI)	p-value	HR (95% CI)	p-value	HR (95% CI)	p-value	HR (95% CI)	p-value
Sex								
Male	1.41 (0.86–2.32)	0.173			1.61 (0.93–2.80)	0.087		
Female	1				1			
Age, median 70								
≥ 70	1.09 (0.67–1.78)	0.726			1.32 (0.76–2.31)	0.328		
< 70	1				1			
Performance status								
1–2	1.89 (1.50–3.12)	**0.012**	1.69 (1.00–2.87)	**0.049**	2.05 (1.19–3.6)	**0.011**	1.86 (1.05–3.32)	**0.033**
0	1		1		1		1	
Smoking status								
Active	2.01 (1.15–3.52)	**0.014**	1.59 (0.87–2.89)	0.128	1.71 (0.92–3.05)	0.079	1.26 (0.65–2.33)	0.478
Never or previous	1		1		1		1	
COPD								
Yes	0.91 (0.52–1.57)	0.732			1.02 (0.79–1.20)	0.846		
No	1				1			
Histology								
Adenocarcinoma	1				1			
SCC	1.27 (0.70–2.28)	0.432			1.34 (0.68–2.50)	0.368		
Other	1.57 (0.66–3.74)	0.304			1.30 (0.80–1.96)	0.238		
PD-L1 tissue								
≥ 50%	1.29 (0.69–2.39)	0.425			1.04 (0.55–2.13)	0.908		
< 50%	1				1			
Line of treatment								
Second or third	0.86 (0.52–1.43)	0.553			0.98 (0.55–1.70)	0.951		
First	1				1			
sPD-L1 baseline								
Numeric values	1.01 (1.00–1.03)	**0.034**	1.01 (0.99–1.02)	0.174	1.02 (1.01–1.03)	**0.006**	1.02 (1.00–1.03)	**0.033**

The same statistical analyses were performed separately on patients receiving pembrolizumab, nivolumab, or atezolizumab as either first line treatment (n = 50) or as second and third line therapy (n = 30) (Supplementary Table 1 and Supplementary Fig. 2). Again, no statistical association was found between sPD-L1 and PFS. Likewise, log-rank test and simple cox regression analysis revealed no relation between sPD-L1 and OS in patients receiving first line therapy. However, when patients treated with the checkpoint inhibitors as second or third line therapy was grouped in tertiles there was a significant association between sPD-L1 and OS (p = 0.040), but this was lost in multivariate analysis adjusting for PS. Due to the low number of patients smoking status was excluded as a covariate in the analysis.

## Discussion

The search for reliable biomarkers to predict the efficacy of anti-PD-1/anti-PD-L1 therapies may result in significant improvements in treatment outcome. Identifying non-responders would allow patients to receive more efficient therapies as well as limit the financial burden of unnecessary treatments^24^. Therefore, former studies in line with our study have tried to elucidate the possible association between sPD-L1 and treatment response of anti-PD-1/anti-PD-L1 therapies in NSCLC patients^12^. The present study demonstrated a weak but significant association between high plasma sPD-L1 levels and shorter OS in NSCLC patients treated with anti-PD-1/anti-PD-L1 therapies in a two-year follow-up. When analyzing the entire follow-up period available this association attenuated -most likely because of confounding variables. Moreover, the data showed a tendency towards sPD-L1 being of prognostic value only in patients receiving second or third line of treatment, however due to lack of statistical power this part should only be considered explorative.

Numerous studies have reported a clear association between high plasma sPD-L1 levels and shorter OS^15,16,20,22^, while others have been unable to detect such link^17,25,26^. Moreover, as Castello et al.^17^ and Yang et al.^26^, we found no significant association between sPD-L1 levels and PFS. Nevertheless, other groups have reported such connection^15,16,20,27^. Furthermore, our results showed no association between sPD-L1 and RR as seen in Okuma et al.^22^ and Costantini et al.^16^. There may be many explanations for these incongruences between the reports. Although the studies seem alike, the cohorts are different both in composition, number of enrolled patients, and duration of follow-up. In our study, 93% of the patients were treated with pembrolizumab but the results presented by Costantini and colleagues came from patients receiving nivolumab therapy. As investigations of sPD-L1 is still in an early stage, it cannot be ruled out that type of anti-PD-1 therapy may influence the results. Likewise, age of the patients, stage and histological subtype of NSCLC, genetics, and line of treatment may be other important factors. Castello et al.^17^ found no association between sPD-L1 levels and OS, PFS or RR, however they reported on a rather small study group, which ought to be sensitive to the potential influencing factors as the ones mentioned. Even in our cohort, there is a tendency towards sPD-L1 being of most value as prognostic marker for OS in patients receiving pembrolizumab, nivolumab, and atezolizumab as second or third line of treatment. This supports a notion that results from the various studies are influenced by the heterogeneity of the cohorts, and thus may be the main reason that results obtained in this area of research are ambiguous. Hence, the most important task will now be to pinpoint for exactly whom sPD-L1 is a reliable biomarker. Moreover, importantly the use of different sPD-L1 assays and materials (serum vs. plasma) across the studies may affect the outcome. In fact, an in-depth analysis of this particular issue will be very valuable if not necessary for understanding the vast amount of sPD-L1 data currently being generated and thus for sPD-L1 to become clinically applicable.

The results from our study suggest that sPD-L1 could be a prognostic marker in NSCLC patients receiving anti-PD-1/anti-PD-L1 therapy, but similar results have previously been obtained for alternative types of treatment. Investigations in patients with advanced NSCLC receiving chemotherapy or best supportive care showed a significant association between high sPD-L1 concentrations and shorter OS^28^. Zhao et al. evaluated the sPD-L1 levels during thoracic radiotherapy in inoperable, locally advanced NSCLC patients, and observed significant longer OS in patients with low baseline sPD-L1 levels^29^. Likewise, Zhang and colleagues found low sPD-L1 levels significantly associated with a longer OS in patients with NSCLC not treated with anti-PD-1/anti-PD-L1 therapies^30^. Collectively, these results indicate that sPD-L1 may be a potential overall prognostic biomarker in NSCLC.

According to our results, sPD-L1 remained rather stable throughout treatment with pembrolizumab or nivolumab. The outcome was the same for patients given the checkpoint inhibitors as first line therapy and second or third line treatment. In contrast, work by Castello et al.^17^ showed a significant increase of median sPD-L1 from baseline and after 3–4 cycles of pembrolizumab or nivolumab therapy, while Oh et al.^21^ found variable concentrations of sPD-L1 pre- and post-treatment in several cancers. As for NSCLC patients specifically, a significant association between longer PFS and OS and an increase sPD-L1 levels was reported by Oh et al.^21^, while Costantini and colleagues found a significant association between shorter PFS and OS and an increase in sPD-L1^16^. Unfortunately, most studies do not have longitudinal measures of sPD-L1, hence further investigations are required to draw any conclusions.

Interestingly, in our cohort, sPD-L1 baseline samples from NSCLC patients receiving anti-PD-1/anti-PD-L1 therapy as their first line treatment were significantly higher than samples from patients receiving their treatment as a second or third line treatment, indicating that former treatments might have an impact on the sPD-L1 (Table 1). Treatment given before second or third line anti-PD-1/anti-PD-L1 therapy were mainly chemotherapy but some patients went through surgery or received radiotherapy. Hence, our data somewhat deviates from the results shown by Vecchiarelli and colleagues, who found a significant increase in sPD-L1 during chemotherapy treatment^25^. On the contrary, Zhao et al. found a significant decrease in sPD-L1 during thoracic radiotherapy^29^. Collectively, these analyses suggest that the types of previous treatments may affect the level of sPD-L1, however such notion needs to be confirmed in a larger study. In this regard, such change may be influential for our results showing a tendency towards sPD-L1 being of prognostic value only in patients receiving second or third line of treatment. However, this part was only explorative due to the lack of statistical power (Supplementary Fig. 2 and Supplementary Table 1). Nevertheless, it is important to be aware that this observation may imply that Fig. 2 and the associated statistical analyses may be biased, as patients in first-line treatment were combined with patients who had received at least one previous line of therapy. We also found that females have a significantly lower level of sPD-L1 before anti-PD-1/anti-PD-L1 therapy than men (Table 1). Similar results were seen by Castello et al., who furthermore reported that females had shorter OS than men, an association we did not find^17^. Besides sPD-L1, we found PS to be a factor of significant prognostic value for OS in the multiple Cox Regression. Constantini et al.^16^ and Muramaki et al.^15^ reported of a similar correlation, though their classification of PS was PS 0–1 and PS > 1 and ours were PS 0 and PS 1–2. Finally, like most other investigations in NSCLC, we were unable to find an association between PD-L1 expression level in tumor tissue and concentration of sPD-L1^16,20,21,27^.

In our study three different anti-PD-1/anti-PD-L1 therapies are represented, pembrolizumab, nivolumab, and atezolizumab. Only three patients received atezolizumab and one of these only got one cycle of treatment and was then excluded because of brain metastases from a cancer mamma. In this case, we only sampled serum at baseline. Interestingly, by use of two different assays, in the two remaining patients we measured extremely high levels of sPD-L1. To the best of our knowledge, this has only been published and commented on once before^31^. Specifically, that study described sPD-L1 as a potential prognostic factor in urothelial cancer patients and, like us, the authors found a 25-fold increase in this potential biomarker during atezolizumab treatment. An increase in the concentration of sPD-L1 in two such different settings indicates that we may be dealing with a yet unknown effect of atezolizumab. However, although these results were obtained using immunoassays from different manufacturers, it is also possible that the extreme high levels of sPD-L1 measured were caused by atezolizumab interfering with the assays. In line with this thought, Oh and colleagues observed a significant rise in sPD-L1 in certain individuals, while others exhibited a stable sPD-L1 level after receiving immune checkpoint inhibitors^21^. A number of the patients included were treated with atezolizumab. Although the authors’ hypothesized that variations in the post therapy levels could be attributed to diverse sources of sPD-L1 or differences in tumor biology, it is possible that the increase observed was linked to the specific checkpoint inhibitor used. Unfortunately, such notion cannot be confirmed from the available data. Hence, for sPD-L1 to become a reliable biomarker in the future, it is of utmost importance to analyze this observation in more detail and therefore experiments are ongoing in our lab to elucidate the matter. However, whatever the outcome of such investigations, our results indicate that type of treatment, sampling time point, and likely also the immunoassay used, need to be taken into consideration when utilizing sPD-L1 as a biomarker.

Our study comes with some limitations. The number of NSCLC patients is limited, especially when grouping them based on lines of treatment. As outlined by Ancel and colleagues in a recent review, unfortunately this is a recurrent issue in many studies describing the potential prognostic role of several promising biomarkers in NSCLC^12^. Furthermore, the absence of chemo-immunotherapy combinatory regimens do not allow definitive conclusions. In addition, the lack of both an external validation cohort and a control group not treated with anti-PD-1/anti-PD-L1 therapies limits the definition of predictive significance of our results. For now, there is no standard technique for measuring sPD-L1 levels, meaning that the sPD-L1 concentrations across studies are not necessarily comparable; Some assays may detect the whole pool of sPD-L1 while others may only measure parts of the total given that sPD-L1 comes in different forms as mentioned previously. Unfortunately, we are not aware of which form the Simoa^®^ PD-L1 Discovery Kit or the Human/Cynomolgus Monkey PD-L1/B7-H1 Quantikine ELISA kit measure, but we are conducting experiments in the lab to uncover this question. Notably, the technical matters have been raised as a general concern and limitation for several promising biomarkers in a recent review describing prognostic biomarkers in NSCLC patients receiving immune checkpoint inhibitors^12^.

In conclusion, this retrospective study suggests that serum sPD-L1 levels may be associated with OS in NSCLC patients treated with anti-PD-1/anti-PD-L1 therapies within a 2-year follow-up period. However, the clinical utility of sPD-L1 measurements in patients treated with anti-PD-L1 calls for further investigations.

### Supplementary Information

Supplementary Figure 1.

Supplementary Figure 2.

Supplementary Table 1.

## Data Availability

The data that support the findings of this study are available from Vejle Hospital—University Hospital of Southern Denmark, but restrictions apply to the availability of these data, which were used under license for the current study, and so are not publicly available. Data are however available from the corresponding author, Line Nederby, upon reasonable request and with permission of Vejle Hospital—University Hospital of Southern Denmark and the Regional Committee on Health Research Ethics in Southern Denmark.
