# Supplementary material for: Soluble programmed death ligand 1 as prognostic biomarker in non-small cell lung cancer patients receiving nivolumab, pembrolizumab or atezolizumab therapy
Source: Sci Rep. 2024 Apr 18;14:8993. doi: 10.1038/s41598-024-59791-0 (PMC11026506; doi:10.1038/s41598-024-59791-0)
Supplement: Supplementary file 1 — Supplementary Figure 1. [file 41598_2024_59791_MOESM1_ESM.pdf]

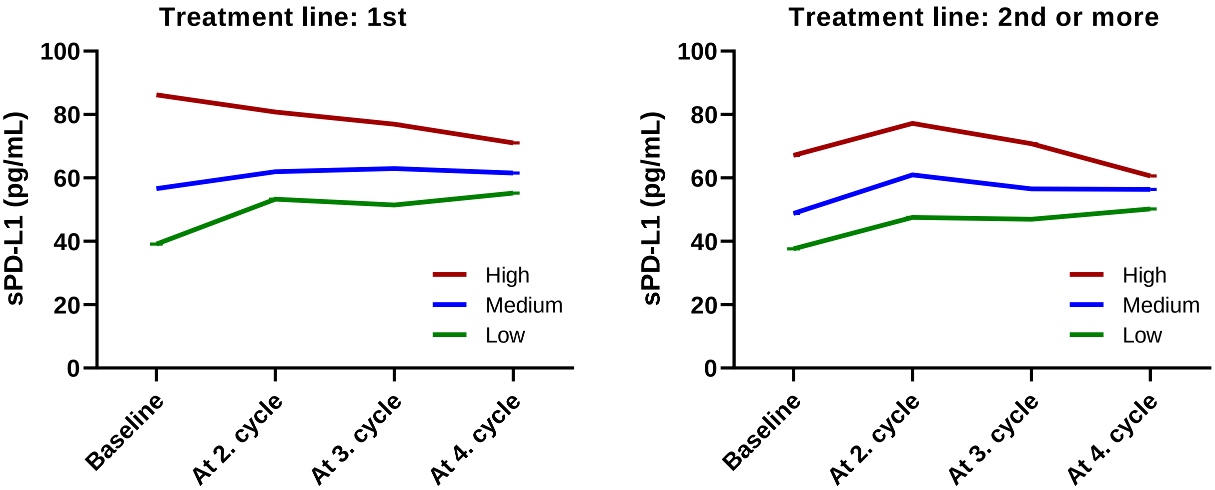

Supplementary Figure 1. Patients were divided in tertiles based on the sPD-L1 baseline level (red=high, blue=medium, green=low). Graphs illustrate the baseline level and dynamics of sPD-L1 in the subsequent samples in the three groups. Left panel shows patients receiving pembrolizumab, nivolumab, or atezolizumab as first line therapy (red, n=16, blue, n=18, and green, n=16), while right panel shows patients receiving it as second or third line treatment (red, n=10, blue, n=10, and green, n=10).
