# Supplementary material for: Soluble programmed death ligand 1 as prognostic biomarker in non-small cell lung cancer patients receiving nivolumab, pembrolizumab or atezolizumab therapy
Source: Sci Rep. 2024 Apr 18;14:8993. doi: 10.1038/s41598-024-59791-0 (PMC11026506; doi:10.1038/s41598-024-59791-0)
Supplement: Supplementary file 2 — Supplementary Figure 2. [file 41598_2024_59791_MOESM2_ESM.pdf]

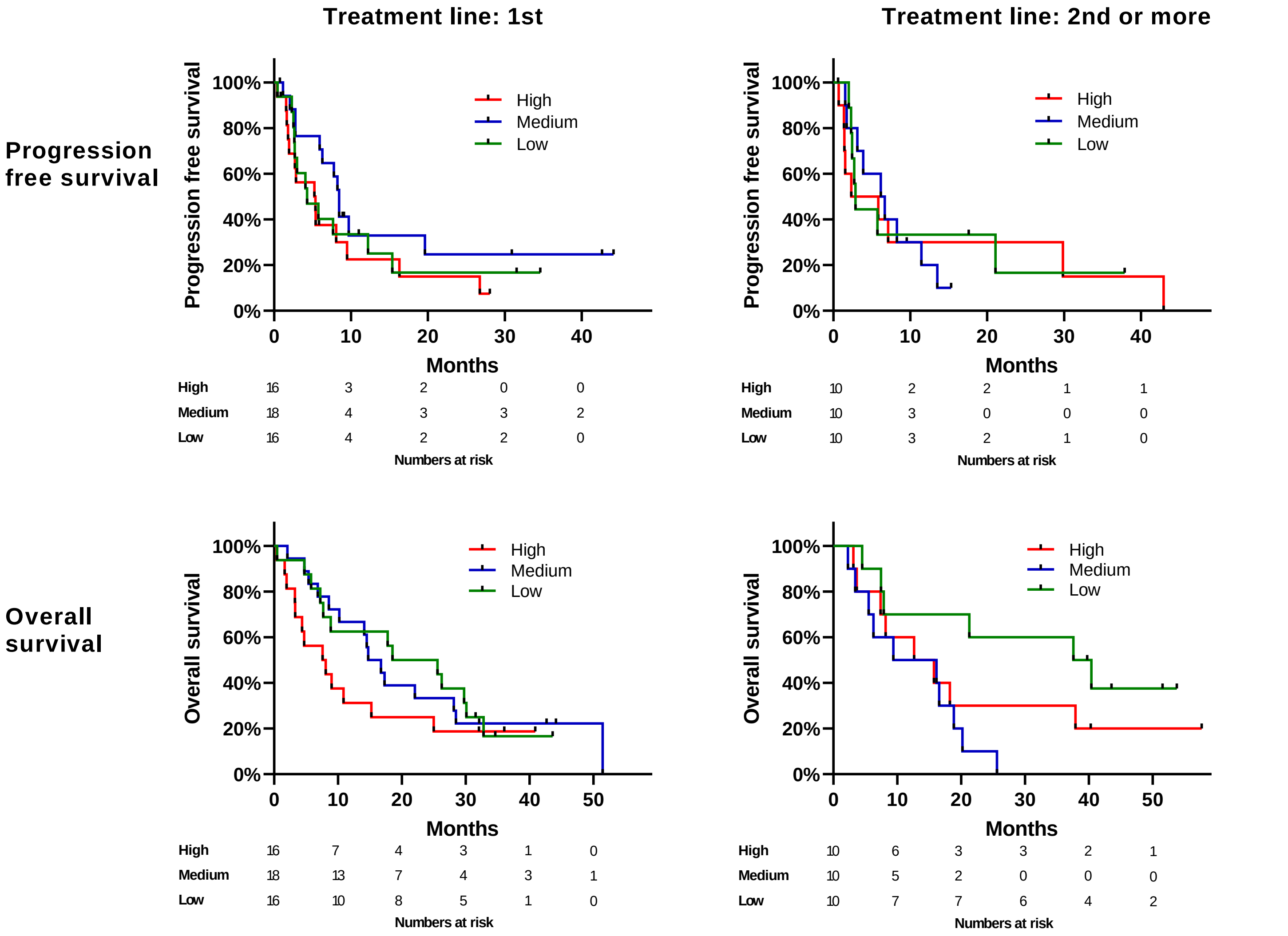


**Supplementary Figure 2**. Progression free survival and overall survival according to baseline levels of soluble programmed death ligand 1 (sPD-L1). Left panels show patients receiving first line treatment (n=50), while right panels are patients given pembrolizumab, nivolumab, or atezolizumab as second or third line therapy (n=30). The patients were divided in tertiles based on the sPD-L1 baseline level (red=high, blue=medium, green=low).
