# Supplementary material for: Soluble programmed death ligand 1 as prognostic biomarker in non-small cell lung cancer patients receiving nivolumab, pembrolizumab or atezolizumab therapy
Source: Sci Rep. 2024 Apr 18;14:8993. doi: 10.1038/s41598-024-59791-0 (PMC11026506; doi:10.1038/s41598-024-59791-0)
Supplement: Supplementary file 3 — Supplementary Table 1. [file 41598_2024_59791_MOESM3_ESM.pdf]

Supplementary Table 1. Log-rank test and Cox regression for patients receiving pembrolizumab, nivolumab, or atezolizumab as either 1st line treatment (n=50) or 2nd and 3rd line treatment (n=30)

**Parameter**	**Log-rank**	**Simple test**		**Multivariate test***	
	p-value	HR (95% CI)	p-value	HR (95% CI)	p-value
**Progression free survival**					
Line of treatment					
First	0.298	0.99 (0.94-1.02)	0.473		
Second or third	0.950	0.96 (0.85-1.04)	0.371		
**Overall survival**					
Line of treatment					
First	0.373	1.02 (0.97-1.07)	0.481		
Second or third	**0.040**	1.02 (0.99-1.05)	0.087	1.46 (0.89-2.43)	0.134
**Progression free survival, 2 years follow-up**					
Line of treatment					
First	0.393	1.01 (0.99-1.02)	0.385		
Second or third	0.981	0.97 (0.86-1.05)	0.539		
**Overall survival, 2 years follow-up**					
Line of treatment					
First	0.143	1.00 (0.97-1.03)	>0.999		
Second or third	0.089	1.02 (0.99-1.05)	0.108		

*Due to lack of statistical power the analysis as performed using only sPD-L1 tertiles and performance status.
